# Hypercalcemia: An Unusual Manifestation of Uterine Leiomyoma

**DOI:** 10.1155/2013/815252

**Published:** 2013-02-12

**Authors:** Amarinder Singh Garcha, Purva Gumaste, Sujith Cherian, Apurv Khanna

**Affiliations:** ^1^Department of Internal Medicine, SUNY Upstate Medical University, 750 East Adams Street, Syracuse, NY 13210, USA; ^2^Division of Nephrology, SUNY Upstate Medical University, 750 East Adams Street, Syracuse, NY 13210, USA

## Abstract

We present a case of hypercalcemia in a 79-year-old female likely secondary to uterine leiomyoma. To the best of our knowledge, hypercalcemia due to a benign tumor has only been described in five cases. Of these above five cases, uterine leiomyoma was thought to be the cause of hypercalcemia in three cases.

## 1. Introduction

Hypercalcemia in the setting of a malignancy is a well-defined entity. Hypercalcemia in association with a benign tumor is very uncommon. We present a case of hypercalcemia in a 79-year-old female likely secondary to uterine leiomyoma.

## 2. Case

A 79-year-old female was brought to the hospital with altered mental status and acute renal failure after a fall. She was lethargic but had no focal neurologic deficits. Laboratory data was unremarkable except for a serum creatinine of 2.8 mg/dL and serum calcium of 17 mg/dL. She was found to have traumatic subarachnoid hemorrhage for which no acute neurosurgical intervention was thought to be necessary. 

 Renal ultrasound, serum, and urine protein electrophoresis were unrevealing. Further workup revealed a suppressed parathyroid hormone level of 14 pg/mL (normal: 15–65 pg/mL), 25-hydroxy vitamin D level of 28 ng/mL (normal >32 ng/mL), and an elevated parathyroid hormone related peptide (PTHrP) 40 pg/mL (normal: 14–27 pg/mL). Due to concerns of a malignancy, the patient underwent CT scans of the abdomen and pelvis which revealed multiple punctate calcifications within the uterus thought to be leiomyomas. A CT scan of the thorax was unrevealing. The patient also received a bone scan which did not reveal any areas of osteolytic metastatic disease. Her CA 19-9 and CEA levels were normal but her CA 125 level was elevated at 48 units/mL (<35 units/mL). A transvaginal ultrasound revealed heterogeneously echogenic uterus with multiple ill-defined lesions suggestive of leiomyomas. This was followed by an MRI of the pelvis without contrast which revealed multiple leiomyomas with the dominant one involving the right lateral wall of the uterus.

Her hypercalcemia and renal failure resolved with aggressive hydration and intravenous furosemide.

## 3. Discussion

Hypercalcemia is seen in nearly 20–30 percent of the patients with a malignancy [1]. This is frequently seen in bone and lung cancers and occurs most commonly due to production of parathyroid hormone-related protein [2]. The PTH-like biological activity of PTHrP is contained within the first 34 amino acids which comprises of the receptor binding and activation domains [3]. PTH and PTHrP have identical actions through a common receptor [3]. Like PTH, PTHrP causes hypercalcemia by promoting bone resorption and decreasing calcium excretion [3].

Osteolytic bone disease caused by secretion of PTHrP also involves stimulation of osteoclasts that in turn secrete tumour-activating transforming growth factor beta which further stimulates local cancer cells in the setting of metastatic disease [2].

PTHrP-mediated hypercalcemia associated with a benign tumor is extremely rare and has been described in uterine leiomyoma, dermoid cysts of the ovary, and mammary hyperplasia [4]. To our knowledge, hypercalcemia associated with uterine fibroids has been described in three cases so far and is thought to be associated with the production of PTHrP [4]. PTHrP is thought to be a normal tissue of female reproductive tract and has important function in late pregnancy and lactation where it may cause vasodilatation and smooth muscle relaxation [5]. The expression of PTHrP has been found to be higher in fibroids than in normal myometrium [5].

In the case discussed above, the patient was found to have elevated calcium levels in the setting of an elevated PTHrP, suppressed PTH, and a low normal vitamin D level. In the absence of a clear focus of PTHrP secretion, uterine fibroids were presumed to be the likely source. In the cases described earlier in the literature, the authors were able to demonstrate resolution of hypercalcemia with hysterectomy and on one occasion had histologic staining positive for PTHrP [4, 6]. It is also worth mentioning that PTHrP has not been shown to regulate 1,25-dihydroxyvitamin D levels independent of serum calcium levels as has been demonstrated by Schilling et al. [7].

Thus, uterine leiomyoma should be considered as a possible cause of hypercalcemia mediated through PTHrP in the absence of any other obvious malignancy.
